# The emerging role of osteoclasts in the treatment of bone metastases: rationale and recent clinical evidence

**DOI:** 10.3389/fonc.2024.1445025

**Published:** 2024-08-01

**Authors:** Youjun Liu, Huanshi Chen, Tong Chen, Guowen Qiu, Yu Han

**Affiliations:** ^1^ Department of Spinal Surgery, Liuzhou Municipal Liutie Central Hospital, Liuzhou, China; ^2^ Department of Orthopedics, The First Affiliated Hospital of Zhengzhou University, Zhengzhou, China

**Keywords:** osteoclasts, bone metastasis, antiresorptive drugs, therapeutic targets, targeted therapy

## Abstract

The occurrence of bone metastasis is a grave medical concern that substantially impacts the quality of life in patients with cancer. The precise mechanisms underlying bone metastasis remain unclear despite extensive research efforts, and efficacious therapeutic interventions are currently lacking. The ability of osteoclasts to degrade the bone matrix makes them a crucial factor in the development of bone metastasis. Osteoclasts are implicated in several aspects of bone metastasis, encompassing the formation of premetastatic microenvironment, suppression of the immune system, and reactivation of quiescent tumor cells. Contemporary clinical interventions targeting osteoclasts have proven effective in mitigating bone-related symptoms in patients with cancer. This review comprehensively analyzes the mechanistic involvement of osteoclasts in bone metastasis, delineates potential therapeutic targets associated with osteoclasts, and explores clinical evidence regarding interventions targeting osteoclasts.

## 1 Introduction

An epidemiological investigation revealed that individuals diagnosed with bone metastasis have a prevalence rate of 5.1%, corresponding to an estimated annual incidence of approximately 18.8 cases per 100 000 bone metastasis diagnoses in the United States from 2010 to 2015 (1). Among individuals aged ≥25 years, lung cancer has the highest prevalence as the main site for *de novo* bone metastases, with a rate of 8.7 cases per 100 000 diagnoses in 2015, followed by prostate and breast primaries, with rates of 3.19 and 2.38 cases per 100 000 diagnoses, respectively (1). In particular, patients with breast cancer have a bone metastasis risk of 73%, whereas those with prostate cancer have a risk of 68% (2). Furthermore, patients with lung cancer have a bone metastasis risk of 30%–40%, whereas those with thyroid cancer have a risk of 60% (3). Malignant tumors often spread in bone tissues, with the bone microenvironment commonly considered a pivotal element in the advancement of bone metastasis. The bone microenvironment comprises a diverse array of cellular entities and a complex extracellular matrix (ECM) (4). The intricate interactions between these constituents and tumor cells contribute to the development of bone metastases. The process of bone metastasis development is complex and well-organized, involving several steps. First, a premetastatic microenvironment is established as a breeding ground for seeding disseminated tumor cells (DTCs). Second, DTCs are extravasated from the circulation to settle within the premetastatic microenvironment. Third, DTCs inhabit the bone niche and become dormant to avoid immune surveillance and antitumor therapy. Finally, DTCs are reactivated from the dormant state to develop into clinically detectable metastases (5). The metastasis of cancer cells to the skeletal system can result in the degradation of bone tissue, onset of pain, and an increased susceptibility to fractures (6). The abovementioned process can be considered a pathological mechanism of bone remodeling, involving a delicate balance between osteoclasts and osteoblasts, ultimately leading to bone restructuring (4).

Osteoclasts have been recognized as a potentially effective therapeutic target in various pathological conditions characterized by bone resorption, including osteoporosis and bone metastasis. Several clinical guidelines have recommended drugs targeting osteoclasts as a primary therapeutic approach for bone destructive diseases (7). Systemic antiresorptive drugs that inhibit osteoclasts can provide the desired symptomatic relief. Furthermore, administering bisphosphonates and denosumab can significantly diminish the occurrence of skeletal-related events (SREs) and mitigate the distress caused by pathological bone resorption, particularly in individuals with osteolytic bone metastases (8, 9). Even in prostate cancer cases where osteoblastic lesions are predominant with a concurrent osteolytic component, antiresorptive medications have been found to effectively mitigate the symptoms associated with bone metastases (10). Nevertheless, antiresorptive medications are currently utilized solely as adjuvant therapies in clinical settings.

**Figure 1:**
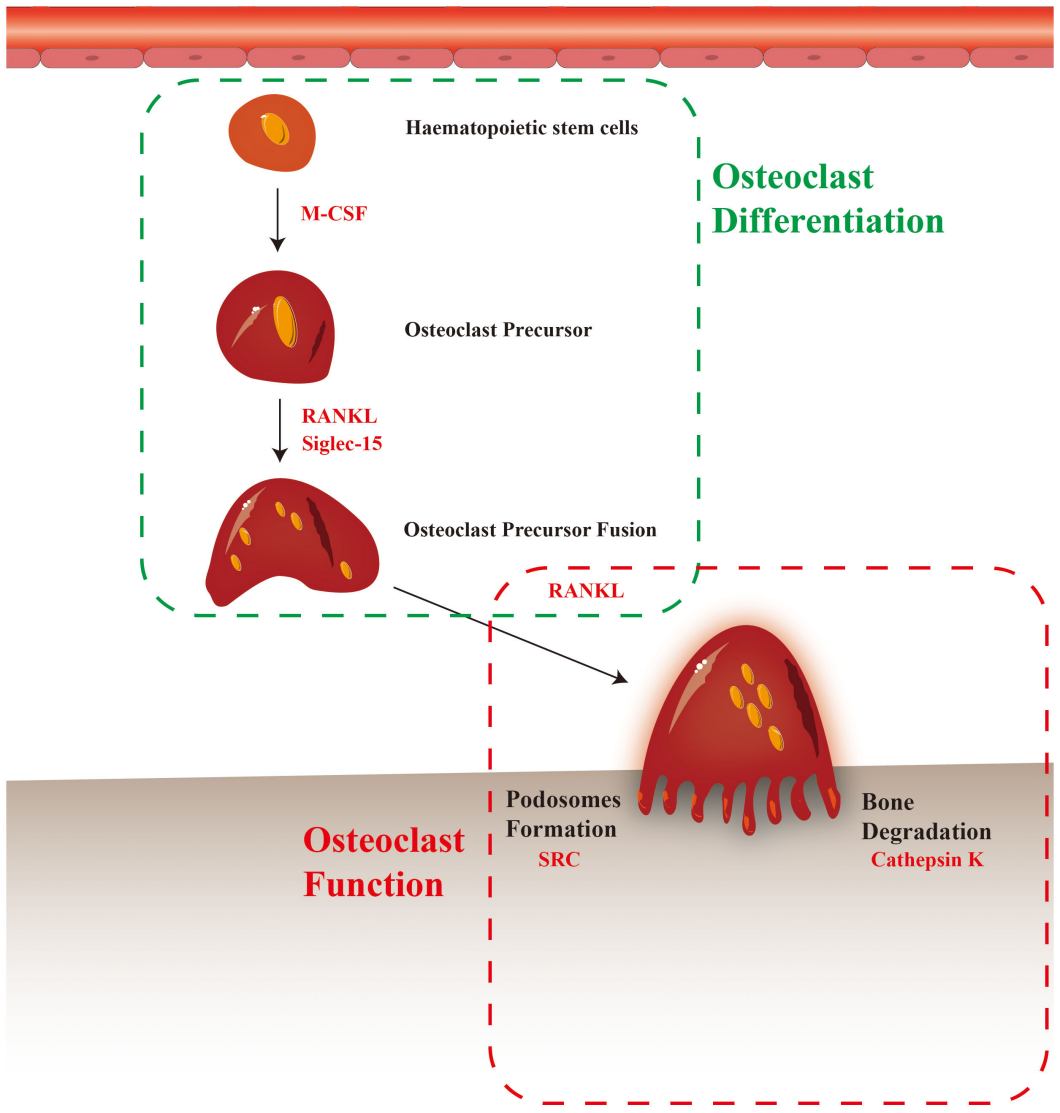
Potential mechanisms of osteoclast involvement in bone metastasis.

**Figure 2:**
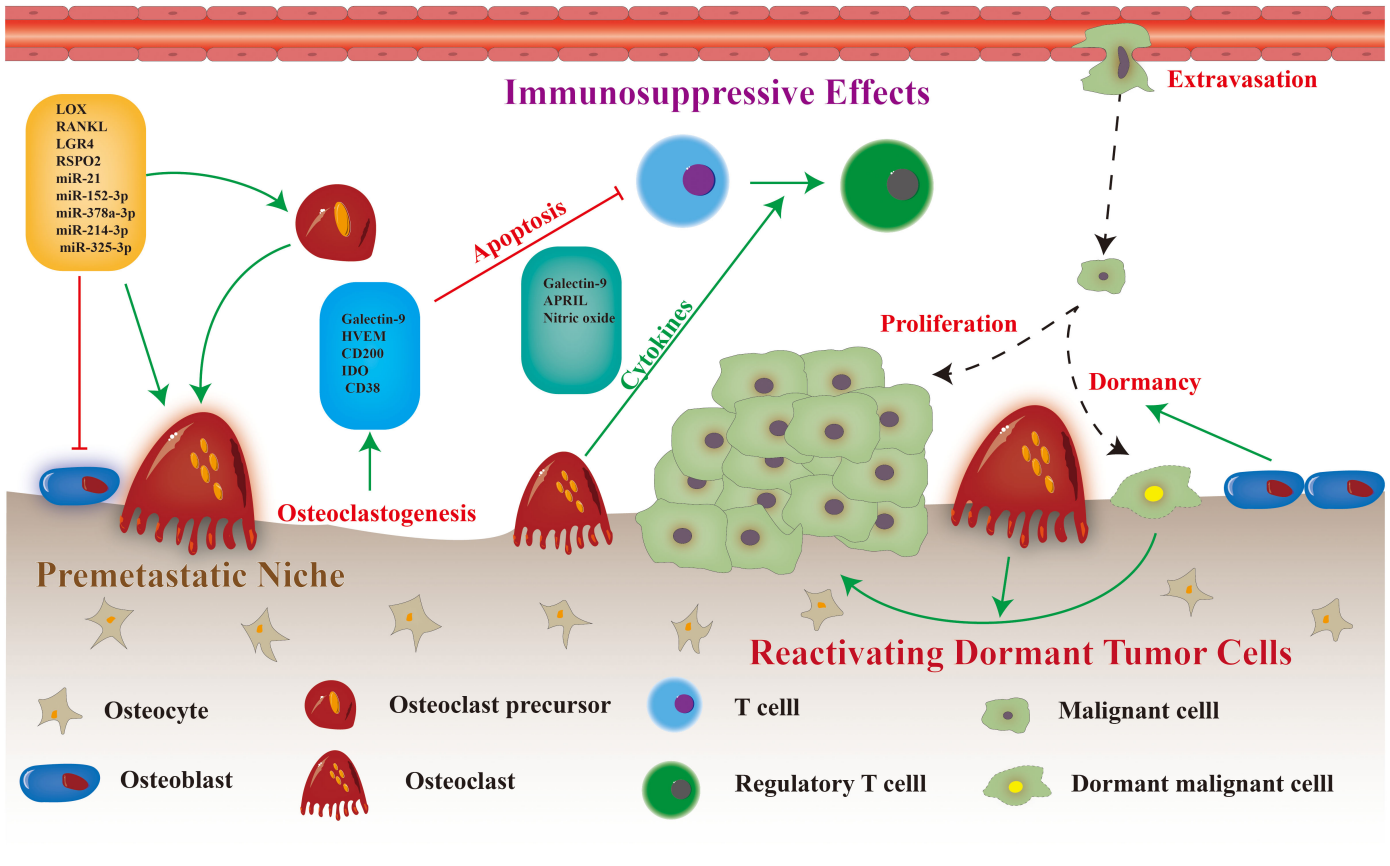
Osteoclast differentiation and maturation, and potential therapeutic targets (the red text in dashed box).

Osteoclasts contribute significantly to the progression of bone metastasis, as they facilitate the formation of the premetastatic microenvironment, modulate immunity to promote tumor cell immune evasion, and stimulate the proliferation of dormant tumor cells (**Figure 1**). Various potential targets for regulating the differentiation and maturation of osteoclasts have been proposed in preclinical studies (**Figure 2**) (11). The efficacy of corresponding inhibitors or monoclonal antibodies for these targets against bone metastases has been demonstrated in animal models (12). However, most clinical trials have yielded inconclusive results regarding the effectiveness of osteoclast-targeted interventions in the treatment or prevention of bone metastases, with the exception of postmenopausal patients with breast cancer (13–15). The precise mechanism underlying bone metastasis remains elusive, and the precise involvement of osteoclasts in this process remains unclear. Recent single-cell studies have revealed that the heterogeneity of osteoclast phenotypes is significantly greater than previously assumed (16). These knowledge gaps may explain the differences between preclinical research findings and their clinical translations. Thus, this review aimed to summarize recent research findings regarding the precise mechanisms of osteoclasts in bone metastasis. Additionally, the review focused on recent advancements in identifying therapeutic targets for osteoclasts and analyze the current status of clinical trials involving relevant drugs.

## 2 Roles of osteoclasts in bone metastasis

### 2.1 Fostering the premetastatic niche

The premetastatic microenvironment refers to the structural and physicochemical alterations in distant organs that facilitate the metastatic expansion of a tumor before tumor cell colonization (17). Numerous mechanisms within bone tissue promote the establishment of a premetastatic microenvironment, both in the normal and pathological states (18). As bone metastases are mostly osteolytic, osteoclasts and osteoclastogenesis are considered significant in the development of the premetastatic niche. The activation of osteoclasts by external stimuli triggers a series of reactions involving vascular modifications, immunological modulation, and metabolic alterations (19, 20). Notably, previous research has revealed the presence of osteoclast-mediated osteolytic foci preceding the metastasis of neoplastic cells to osseous tissue (21). Furthermore, besides their involvement in bone tissue, osteoclast precursors and monocytes in the circulation and primary tumor sites can induce peripheral osteoclastogenesis, facilitating the progression of bone metastases through multiple pathways.

The development of bone metastases in breast cancer cases, characterized by osteolytic changes, is frequently facilitated by the manipulation of the premetastatic microenvironment by osteoclasts. According to a previous study, breast cancer cells with a propensity for bone metastasis can secrete S100A4 protein. This protein has been found to play a significant role in promoting osteolysis by directly stimulating osteoclast formation through the surface receptor RAGE. Breast cancer cells can also release R-spondin 2 and receptor activator of nuclear factor kappa-B ligand (RANKL), which are essential proteins for attracting osteoclast precursors and fostering the development of the osteoclastic premetastatic niche (22). Furthermore, tumor cells can control osteoclast precursors, as evidenced by a study demonstrating that HCC cells secreting lectin galactoside‐binding soluble 3(LGALS3) can induce the fusion and podosome formation of osteoclasts through the CD98–integrin αvβ3 complex, resulting in osteolytic bone remodeling (23). However, the premetastatic bone microenvironment responds differently to LGALS3 released by different tumor cells. LGALS3 can enhance osteoclast differentiation in breast cancer, whereas in prostate cancer, it may only affect osteoblast development without affecting osteoclast differentiation.

In addition to protein secretion, neoplastic cells can discharge exosomes and microvesicles that carry diverse pro-oncogenic molecular cargo, thereby impacting bone homeostasis and forming the premetastatic microenvironment. A previous study revealed that SCP28 breast cancer cells release exosomal miR-21, which can bind to programmed cell death 4 in osteoclasts, thereby stimulating osteoclast differentiation and intensifying bone metastasis (21). Our study findings indicate that patients with breast cancer who have developed bone metastases exhibit higher levels of blood exosome miR-21 than those who have not developed this condition. This finding suggests that miR-21 can potentially serve as an early detection marker for breast cancer bone metastases (21). Osteoblastic bone metastases, including those originating from prostate cancer cells, can regulate osteolysis and hinder osteogenesis through exosomes, thereby promoting the advancement of bone metastasis in tumor cells (24). Several studies have demonstrated that tumor-derived extracellular vesicles containing diverse microRNAs (miRNAs), including miR-152–3p, miR-378a-3p, miR-214–3p, and miR-325–3p, can modulate osteoclast differentiation (25–28). Furthermore, research has shown that exosomal miRNAs are potential indicators of the progression of bone metastases (29). miRNAs hold significant potential in the advancement of novel diagnostic techniques and therapeutic interventions for bone metastases. Previous study has revealed that MRX34, an miR-34 mimic currently undergoing clinical trials for treating hepatocellular carcinoma, protects against the development of osteolytic disease in individuals with metastatic breast cancer (30). However, the use of miRNAs as therapeutic agents for bone metastases presents various challenges, such as suboptimal delivery efficacy and undesired immune responses, which necessitate further investigation (29).

In addition to directly acting on osteoclasts, exosomes can be taken up by myeloid cells in the bone marrow, eliciting various premetastatic responses, including stimulation of NF-κB signaling, accelerated development of osteoclasts, and decreased expression of myeloid thrombospondin-1 (31). The activation of osteoclasts induces a series of osteolytic reactions, resulting in an intensified local inflammatory response. Finally, these inflammatory responses induce changes in vascular permeability and promote the extravasation process of DTCs (32).

Various researchers have effectively cultured osteoclasts from diverse bone marrow and circulating myeloid populations, such as monocytes and dendritic cells (DCs), in humans and animals (33). Emerging evidence indicates that the migration of osteoclast precursors away from the bone considerably influences the development of the premetastatic niche in bone metastasis. In the field of bone metastases, the mechanism through which myeloid-derived suppressor cells (MDSCs) serve as progenitors to osteoclasts has attracted significant attention. The proportion of MDSCs in the peripheral blood of patients with breast cancer is approximately 10 times that of healthy controls (34). A strong correlation exists between heightened numbers of circulating MDSCs and the progression of cancer to an advanced stage, as well as the presence of a positive lymph node status (34). MDSCs can express specific integrins, chemokines, and ECM regulatory factors, thereby exerting influence on vascular permeability, modifying the composition of the ECM, recruiting various cell types, suppressing immune responses, and facilitating the survival of DTCs in the bloodstream as well as their migration to distant metastatic sites (35). Upon infiltration of tumor cells into bone metastases sites, MDSCs undergo differentiation into active osteoclasts, facilitating the expansion of osteoclastic lesions (36). The monocyte–macrophage lineage is a significant progenitor of osteoclasts and is found extensively in the tumor and bone microenvironments, as well as in the circulatory system. Similar to the function of MDSCs, the monocyte–macrophage lineage can not only influence the progression of bone metastases through mechanisms such as immune modulation and vascular permeability alteration but also differentiate into osteoclasts under specific circumstances (37). DCs have been recognized as a plausible origin of osteoclast precursors during inflammation. The differentiation of osteoclasts from DCs may serve as an alternate mechanism for osteoclast formation in the bone premetastatic niche. Splenic CD11c+ DCs were effectively induced in mature and activated multinucleated giant cells expressing TRAP and IL-23 when exposed to conditioned media from breast cancer cells (38).

### 2.2 Immunosuppressive effects

Once DTCs infiltrate bone tissue, the first challenge is whether they can successfully survive immune system surveillance. Several pieces of evidence have demonstrated the involvement of osteoclasts in the immunological regulation of bone metastases. The *in vitro* expansion of natural killer (NK) cells using osteoclasts from patients with cancer resulted in a significant reduction in the quantity and cytotoxicity of NK cells. However, introducing osteoclasts from healthy individuals restored the cytotoxic activity of NK cells (39, 40). The dissimilarities observed in osteoclasts from individuals with tumors and those without tumors suggest that osteoclasts can attenuate immune responses under pathological conditions, thereby preventing the immune system from effectively targeting tumor cells. This section summarizes how osteoclasts contribute to the immunosuppressive impact of bone metastases, including the direct and indirect regulation of cytotoxicity.

The abovementioned clinical finding supports the theory that osteoclasts are immune-competent cells. Multiple *in vitro* investigations have suggested that osteoclasts can directly influence the state of T or NK cells through cell-contact–independent mechanisms. Osteoclasts can directly suppress proliferation and induce apoptosis of T cells by secreting Galectin-9 and CD200, creating an immunosuppressive environment in several patients with myeloma (41). *In vitro* empirical investigations have further confirmed that osteoclasts secrete various cytokines that mitigate the activation, proliferation, cytotoxic activity, and apoptosis of T cells (42). The inactivation of T cells can facilitate osteoclast formation in bone metastases, thereby exacerbating the vicious cyclic progression of bone metastases (43). In addition to directly affecting T cell function, osteoclasts can influence immune activity within bone metastases by regulating the expression of immune checkpoint molecules. A prominent illustration of this phenomenon is the capacity of osteoclasts to facilitate an immunosuppressive environment by secreting a proliferation-induced ligand (APRIL), resulting in enhanced programmed cell death ligand-1(PD-L1) expression in multiple myeloma cells (41). Several studies have established a strong correlation between the expression of immune checkpoints in bone metastases and osteoclasts (44, 45). Furthermore, the regulation of osteoclast activity may be influenced by immune checkpoint expression (41).

Osteoclasts can establish an immunosuppressive microenvironment by interacting with immunomodulatory and immunosuppressive cells, in addition to directly influencing the activity of T and NK cells. Furthermore, osteoclasts contribute to the immunological tolerance of malignancies by inducing regulatory T cell responses (19). Bone marrow-derived inflammatory osteoclasts (Cx3cr1+) secrete immunosuppressive cytokines and polarize CD4+ T cells into immunosuppressive CD4+ Foxp3+ regulatory T cells in an antigen-dependent manner (46). Another study revealed that PD-L1 is an essential component of the immunosuppressive capacity of Cx3cr1+ osteoclasts and that Cx3cr1 can distinguish between two different subsets of osteoclasts, each with a distinct role in the immune system (47). Moreover, osteoclasts can attract and activate naïve CD8 T cells, resulting in the transcription of CD25 and Foxp3 genes (48).

MDSCs are widely recognized as immunosuppressive cells that can suppress T cell function. As precursors of immature myeloid cells, MDSCs can differentiate into macrophages and osteoclasts, enabling the regulation of bone resorption under various pathological conditions (49). Consequently, they are considered osteoclast precursors. A previous revealed that the accumulation of MDSCs in bone metastases may decrease the effectiveness of bisphosphonates (50). The interaction between MDSCs and osteoclasts holds relevance in the context of bone metastasis (51). However, the existing literature on the modulation of MDSCs by osteoclasts to facilitate immune evasion in bone metastases is limited, necessitating further investigation.

However, certain studies have proposed that osteoclasts can potentially enhance immune responses. As antigen-presenting cells, osteoclasts express both Class I and Class II of the major histocompatibility complex (MHC). Furthermore, they can take up soluble antigens, facilitating the presentation of allogeneic antigens, which, in turn, induce the activation of CD4+ and CD8+ alloreactive T cells in a manner restricted by the MHC (52). However, there is no supporting evidence for the alteration of antigen-presenting activity in osteoclasts throughout bone metastasis. Bone metastases alter the antigen-presenting capacity of DCs, which are osteoclast precursors. In breast cancer animal models, the percentage of plasmacytoid DCs(pDCs) that infiltrated bone metastases was significantly higher, but their antigen-presenting ability was considerably diminished compared to other DCs subtypes (53). Furthermore, the cytotoxic activity of T cells was significantly restored following pDCs depletion.

### 2.3 Reactivating dormant tumor cells

Metastases may occur at any time, even after the surgical removal of the primary tumor. This delayed metastasis, also known as relapse, is attributed to mechanisms that maintain DTCs in a quiescent or latent state where they cannot proliferate (referred to as “tumor dormancy”) (54). The dormancy of malignant cells can be reversed or transitioned between “on” and “off” states by specific signals from the surrounding microenvironment (55). The interaction between cancer cells and their bone microenvironment plays a critical role in regulating cellular dormancy and reactivation. Research has suggested that osteoblasts can induce metastatic tumor cells into a dormant state by secreting growth factors, thereby preventing their elimination via antitumor therapies (56). Understanding the mechanisms underlying tumor dormancy and its reactivation is crucial in preventing metastatic advancement and extending metastasis-free survival. However, the molecular and cellular processes responsible for activating dormant tumor cells remain poorly understood.

A subset of DTCs are located in the endosteal niche, where they enter a quiescent state characterized by G0–G1 cell cycle arrest (55). Some of these DTCs remain in a quiescent state, whereas others continue to multiply and form micrometastases. However, actively proliferating DTCs can be eliminated by the immune system or antitumor therapies, whereas dormant DTCs can persist in the endosteal niche with the potential for reactivation, which can lead to disease relapse (10). Once DTCs reach the bone tissue, they compete with HSCs for the osteoblastic niche and eventually colonize the compartment previously occupied by HSCs (57). Additionally, osteoblasts protect dormant DTCs from environmental stress stimuli through paracrine and juxtacrine secretion (58). Recent studies using single-cell level analysis have further supported the notion that osteoblasts play an essential role in inducing and protecting dormant DTCs, both *in vitro* and *in vivo* (59–61). For prostate tumors, the production of Wnt5a in the osteoblastic niche can activate noncanonical ROR2/SIAH2 signaling and induce dormancy in DTCs (62). Recent gene array analyses have identified GDF10 and TGF2 as osteoblast-secreted proteins that induce quiescence in certain prostate tumor cell lines, indicating varying responses to dormancy across prostate cancer cells (60). Furthermore, breast cancer cells interacting with spindle-shaped N-Cadherin+ osteoblasts are recognized and induced into a dormant state through a Notch2-dependent process (63).

The transition from a dormant to active state is a critical event in tumor relapse and metastasis. Osteoclasts play a crucial role in bone remodeling and can directly reactivate dormant DTCs by modifying the endosteal niche. For example, myeloma cells can become dormant through direct contact with osteoblasts in the bone marrow niche, but they can be released from this state when the niche is modified in an osteoclast-dependent manner (61). A longitudinal imaging study using intravital two-photon microscopy revealed that dormant myeloma cells could be reactivated by activating osteoclasts with RANKL (61). Another study demonstrated that the combination of VCAM-1 and integrin α4β1 recruits osteoclast progenitors (OPs), inducing a vicious cycle of bone degradation and tumor progression (64). Suppressing osteoclast activity may reduce the risk of dormant tumor cell reactivation and prevent the development of macrometastatic lesions. Dormancy in cancer cells is a severe clinical issue that directly impacts tumor drug resistance and relapse. Thus, targeting tumor dormancy to prevent tumor metastasis and recurrence is an emerging research area.

## 3 Heterogeneity of osteoclasts and bone metastasis

Bone metastases exhibit a lower incidence in distal extremities but are more prevalent in bones with red marrow and trabecular bone, such as the pelvis, vertebrae, and ribs (5). The underlying mechanisms behind site-specific bone metastases are currently unclear. Some hypotheses suggest that differences in trabecular bone structure, bone turnover rates, and vascularization at these sites may contribute to metastasis formation (65). Currently, single-cell transcriptome techniques are being employed to identify variations in bone homeostasis across different bone regions and under various pathological conditions (16). Additionally, heterogeneous osteoclasts have been identified in different bone sites and under various pathological conditions (16).

Osteoclasts can be classified based on their anatomical location (e.g., calvarial osteoclasts, odontoclasts, and vascular-associated osteoclasts) and their association with certain diseases (e.g., arthritis-, obesity-, and fracture-associated osteoclasts) (16). In a murine model, long bone osteoclasts display a more advanced osteoclastic phenotype than calvarial osteoclasts, with a higher expression of membranous and secreted osteoclast proteins (66). Furthermore, osteoclasts from the cranium and appendicular skeletons have distinct characteristics. This heterogeneity in osteoclasts from different sites is reflected in their osteoclastic activity and bone turnover as well as in their response to therapeutic interventions. For example, osteoclasts from various anatomical locations exhibit different responses to bisphosphonate therapy, which is considered one of the mechanisms underlying bisphosphonate-induced osteonecrosis of the jaw (67).

Currently, the scientific literature on the heterogeneity of osteoclasts across different skeletal locations is limited. Several studies have indicated that this osteoclast diversity can be attributed to the regulation of osteoclast development by various cells within the bone, exhibiting site-specific and disease-dependent patterns. One study found that the RANKL/OPG ratio and TNF-α gene expression were significantly higher in cranially isolated osteoblasts than in long bones, yielding a different process of osteoblast-induced osteoclast formation in different bones (68). A single-cell investigation of osteoclastogenesis in bone marrow revealed that, alongside osteoblasts and osteocytes, marrow adipogenic lineage precursors (MALPs) are vital in osteoclast formation (69). The key role of MALP-derived RANKL in bone remodeling at various skeletal sites was confirmed in adipocyte-specific RANKL-CKO mice (69). The absence of MALPs within the periosteum indicates that RANKL-CKO mice exhibit reduced bone loss, specifically in the trabecular bone, whereas cortical bone remains unaffected. However, the potential variations in MALPs within bone tissue across distinct anatomical locations remain unexplored. Other studies have suggested that variations in inflammatory signaling pathways, such as IL-1 and TNF-α, within different skeletal sites may contribute to phenotypic differences in osteoclasts (70, 71).

Based on statistical findings from clinical data, bone metastases most frequently occur in the spine, with the pelvis being the subsequent site of occurrence (72). While the correlation between the spread of bone metastases and anatomical characteristics is evident (73), the potential influence of variations in cellular composition within the skeletal system remains uncertain. Furthermore, although osteoclasts vary between anatomical sites, the relationship between osteoclast heterogeneity and the predilection of tumor metastases for specific bone sites has not been well researched. Thus, further research is required to ascertain the precise role of osteoclasts in bone metastasis.

## 4 Therapeutic targets of osteoclasts in preclinical studies

### 4.1 RANKL

RANKL is a TNF superfamily member generated by osteoblasts, T cells, and stromal cells (74). When RANK binds to RANKL, several genes and pathways involved in osteoclast growth are activated, including NF-κB, mitogen-activated protein kinase, protein kinase C, and Src kinase (75). Extensive research has highlighted the importance of RANKL in bone remodeling and osteoclast development. RANKL-targeted therapy is one of the most effective therapeutic strategies and is currently used to treat osteoporosis, giant bone cell tumors, and bone resorptive disorders.

RANKL is a type II transmembrane protein with a carboxy-terminal extracellular domain, which is cleaved by proteases, leading to the release of soluble RANKL into the extracellular environment (74). Recent research has shown that membrane-bound RANKL is primarily responsible for regulating musculoskeletal and immune functions in a physiological context, whereas soluble RANKL plays a key role in pathological processes such as bone metastasis formation (76). Additionally, soluble RANKL has been demonstrated to be nonessential in physiological processes; however, it facilitates the spread of cancer to the bone by directly inducing tumor cell migration toward the bone. Notably, the absence of soluble RANKL does not impact osteoclasts at the metastatic site or the dissemination of cancer to nonskeletal organs. Based on these findings, targeting soluble RANKL alone could be a highly specific approach for treating bone metastasis (76).

The ability of osteoclasts to reactivate dormant DTCs can be suppressed by treatment with OPG-Fc, which disrupts *in vivo* RANK–RANKL interactions (77). Additionally, RANKL acts as a mediator in the interactions between the skeletal and immune systems. Various inflammatory factors can activate RANKL, promoting osteoclastogenesis activity in metastatic lesions (78). Modulating the immune environment of tumors has been reported to decrease osteoclast and osteoclastogenesis activities. Within the local tumor microenvironment, the expression of PD-L1 and CCL2 is increased, with PD-L1 facilitating RANKL-induced osteoclastogenesis through JNK activation and CCL2 release (79). In a murine model of bone metastasis, treatment with the PD-1 monoclonal antibody nivolumab inhibited osteoclast development (79). Further research is warranted to determine the role of RANKL in immunotherapy and the mechanism by which RANKL-targeted medications impact the immunological tumor microenvironment.

### 4.2 M-CSF

M-CSF, also known as colony-stimulating factor 1 (CSF-1), plays a vital role in osteoclast differentiation in various stages of development. M-CSF promotes the survival and proliferation of OPs by binding to its receptor, CSF1R. Moreover, M-CSF can restore osteoclast deficiency in M-CSF-deficient osteoporotic models (80). It has several isomeric forms and can be expressed as a membrane-bound protein or secreted as proteoglycans and glycoproteins (81). In addition to promoting cell growth, M-CSF protects OPs from undergoing apoptosis (82).

*In vitro* studies have shown that breast cancer cell lines can stimulate osteoblast differentiation by secreting M-CSF, with M-CSF inhibition potentially reducing the risk of osteolytic metastasis (83). Similarly, a mouse model of lung cancer bone metastasis demonstrated that the knockdown of the CSF-1 gene in A549 cells significantly reduced the number of osteoclasts and inhibited the tendency of tumors to metastasize to bone (84). Osteoclasts and macrophages originate from the same progenitors, which require critical lineage signals to differentiate into distinct cell types. Inhibiting CSF-1/CSF1R signaling can also affect macrophages (85). Therefore, in addition to regulating osteoclast differentiation, considerable preclinical and clinical research efforts have focused on the ability of CSF-1/CSF1R signaling to regulate macrophages. As a relationship exists between macrophages and bone metastasis, the potential antibone metastatic mechanism of CSF-1R may not depend solely on osteoclast inhibition.

While no clinical trials are currently focusing on CSF1R inhibitors, particularly for bone metastasis treatment, several studies are exploring CSF1R inhibitors for various cancer types. These studies might pave the way for further research on patients with bone metastases by elucidating the effectiveness and safety of CSF1R inhibitors in humans.

### 4.3 Src

Src, a nonreceptor tyrosine kinase, belongs to a family of proteins that bind to the cytoplasmic surface of cellular membranes (86). Unlike M-CSF and RANKL which control the differentiation of osteoclasts, Src is mainly responsible for regulating osteoclast function. In particular, osteoclasts rely on Src to facilitate the rapid assembly and disassembly of podosomes, which serve as attachment structures to aid the transition between migratory and resorbing stages (87). A study revealed that Src expression is significantly correlated with bone metastasis in various tumors (88). Dasatinib, an Src kinase inhibitor, has been shown to inhibit osteoclast recruitment in a xenograft mouse model of breast cancer. In a previous study, bioluminescence scan revealed that dasatinib significantly reduced skeletal metastases in triple-negative breast cancer *in vivo*, whereas µ-CT indicated a substantial increase in bone volume in the dasatinib-treated group (89).

The phosphorylation state of Src is influenced by multiple variables. Controlling these variables that regulate Src phosphorylation may limit bone metastasis by suppressing osteoclasts. Macrophage-stimulating protein signals can promote Src phosphorylation by binding to its corresponding receptor, RON tyrosine kinase. RON kinase inhibitors have been employed in preclinical investigations to alter Src phosphorylation in osteoclasts for treating bone metastasis (90). Moreover, factors that may reportedly influence osteoclasts through Src include CXCL12, CCR5, ICAM-1, CX3CL1, and CXCL17 (91–94). These findings demonstrate that Src, similar to RANKL, acts as a link between bone and immune systems and plays an important role in bone metastasis.

### 4.4 Cathepsin K

Cathepsin K, a cysteine protease highly expressed in osteoclasts, belongs to the cathepsin lysosomal protease family. It acts as an osteolytic enzyme and protease, breaking down bone matrix proteins (95). It is closely associated with osteoclast-mediated bone destruction during tumor metastasis. Researchers have recently developed a system for imaging probes (Osteoadsorptive Fluorogenic Sentinel imaging probe) that can respond to osteoclast-secreted cathepsin K and detect osteolytic reactions at the metastatic site of multiple myeloma in a mouse model (96). Preclinical studies have shown that the inhibition of cathepsin K significantly decreases the ability of prostate cancer cells to induce osteoclast-mediated bone resorption, thus decreasing the risk of bone metastasis both *in vitro* and *in vivo* (95, 97). Similar results have been reported in preclinical studies on breast, lung, and renal tumors (98). One such study revealed that the inhibition of cathepsin K may directly affect the function of osteoclasts without altering their number (99). Odanacatib, a cathepsin K inhibitor, does not alter the number of osteoclasts but inhibits the mRNA expression of secreted osteolytic factors, such as PTHrP, CXCR-4, and TNF-α (99).

In addition to osteoclasts, cathepsin K expressed by tumor and other cells within the tumor microenvironment can promote bone metastasis. In human colorectal cancer tissues, the overexpression of cathepsin K is consistently associated with increased M2 tumor-associated macrophages (TAMs) in the stroma, which is related to tumor metastasis and poor prognosis. A recent study proposed that cathepsin K secreted by tumor cells binds to TLR4, leading to the M2 polarization of TAMs through an mTOR-dependent mechanism (100).

### 4.5 mTOR

The mTOR pathway is crucial in regulating skeletal development and homeostasis due to its impact on osteoblasts, osteoclasts, and chondrocytes (101). Although the precise mechanism by which mTOR regulates osteoclasts is being debated, the importance of mTOR signaling in osteoclastogenesis and osteoclast viability has been established. It is widely postulated that several pathways associated with mTOR can influence osteoclastogenesis through diverse mechanisms. Experimental evidence supports the notion that mTOR-mediated restriction Akt signaling regulates osteoclast fusion, whereas the regulation of mTOR-raptor protein translation leads to the cytoplasmic development of individual osteoclasts, independent of fusion (102). Furthermore, mTOR complex 1 (mTORC1) plays a crucial role in modulating bone resorption and maintaining bone homeostasis in pathological scenarios due to its expression in osteoclasts (103). mTORC1 was observed to negatively regulate the expression of NF-κB and NFATc1, which are crucial transcription factors in the differentiation of osteoclasts within osteoclast lineages (104). The mTOR pathway plays a pivotal role as a signaling mechanism that restricts osteoclastic differentiation, suggesting the potential therapeutic application of mTOR inhibitors in managing bone loss–related disorders. Preclinical investigations have reported that the osteoclast population and osteolysis associated with experimental metastases were significantly reduced upon administering Rapamycin (105, 106).

### 4.6 Siglec-15

The activation of adaptor–receptor complexes of the immunoreceptor tyrosine-based activation motif elicits costimulatory signals that contribute to osteoclastogenesis. Molecules such as cytotoxic T lymphocyte-associated antigen 4 for Fc receptor gamma and Siglec-15 for 12-kD DNAX-activating protein have been identified as prospective candidates for inducing alteration in the suppression of osteoclasts. Siglec-15 is an essential immune suppressor that exhibits varying overexpression across many cancer types, making it a promising candidate for cancer immunotherapy (107).

Siglec-15, a member of the Siglec family, is highly conserved and expressed on osteoclasts, some myeloid cells, and certain cancer cells (108). Research has revealed that Siglec-15 is closely involved in osteoclast differentiation and that conditional knockout of Siglec-15 in mice or treatment with Siglec-15–neutralizing antibodies inhibits the multinucleation process of osteoclasts (109). Siglec-15 is also closely linked to intratumor immunity. Therefore, it has been suggested that Siglec-15 not only regulates osteoclasts in the vicious cycle of bone metastasis but also promotes bone destruction through synergistic effects with TGF-β (110). Dou et al. investigate the role of sialylation of TLR2 in osteoclast fusion, demonstrating that Siglec15 binds sialylated TLR2 to activate cell recognition and fusion in osteoclast formation, with implications for bone resorption regulation (111). In subsequent mouse animal model studies, it was discovered that apoptotic bodies derived from osteoclasts in the bone microenvironment inhibit the activation of CD8 T cells through Siglec15, thereby promoting the metastasis of breast cancer (112). Conversely, the use of anti-Siglec15 therapy reduces the occurrence of secondary metastases and enhances survival rates in cases of breast cancer bone metastasis. However, the detailed molecular processes regulating Siglec-15 expression remain unclear, and there is a lack of relevant preclinical studies confirming the effectiveness of Siglec-15–targeted therapy in inhibiting bone metastasis. In addition, Siglec-15 may have a regulatory effect on osteoblasts; however, further in-depth studies are warranted to confirm these findings.

Siglec-15, a molecule sharing 30% structural similarity with the B7 family (PD-L1/B7-H1), can induce immune-regulatory responses by inhibiting the activation and proliferation of T cells through an IL-10-dependent mechanism (113). Furthermore, no apparent correlation was observed between Siglec-15 and PD-L1 expression, indicating that Siglec-15 might have a significant immunosuppressive function independent of PD-L1 (114). Therefore, Siglec-15 could potentially serve as an additional therapeutic target for managing malignancies with resistance to anti-PD-L1/PD-1 immunotherapy.

## 5 Clinical trials focusing on osteoclast-targeted therapy

The selection of suitable treatment approaches for patients with bone metastases relies on various factors, including the distinct attributes of the primary malignancy, presence or absence of metastases in areas other than the skeletal system, and extent of bone involvement (whether it is confined to a specific area or spread throughout the body) (6). Management of bone metastases requires a comprehensive strategy that incorporates several therapies, such as chemotherapy, radiation therapy, biologically targeted agents, endocrine interventions, and surgical procedures. Osteoclast-targeted therapy strategies are a valuable adjunctive treatment for effectively managing SREs, thus providing a crucial complement to existing therapeutic approaches.

The efficacy of antiosteoclastogenic treatment modalities such as bisphosphonate medications and denosumab in managing bone metastases depends on the inherent attributes of the primary tumor. The present focus of clinical trials is on bone metastases originating from breast and prostate cancer, which have shown promising outcomes. However, in case of bone metastases originating from other tumors, these medications are employed solely to control SREs or as a strategy for symptomatic treatment.

### 5.1 Bisphosphonates

Bisphosphonates are widely used antiresorptive medications in clinical practice that have demonstrated promising outcomes in treating osteoporosis and Paget’s disease of the bone. Their efficacy in preventing SREs has been reported in individuals with metastatic bone cancers, such as breast and prostate cancer. Bisphosphonates predominantly exhibit an affinity toward bones at sites of active bone metabolism and dissolve from the bone matrix during bone resorption. They are then taken up by osteoclasts, limiting their activity and survival and mitigating osteoclast-mediated bone resorption (115). Notably, several bisphosphonate medications have unique mechanisms of action on osteoclasts. Non-nitrogen–containing bisphosphonates such as etidronate induce osteoclast apoptosis by forming a toxic adenosine triphosphate analog. Conversely, nitrogen-containing bisphosphonates such as alendronate, zoledronic acid, and ibandronate target the enzyme farnesyl diphosphate synthase, which is necessary for osteoclast function (116).

Bisphosphonates have been demonstrated to minimize the risk of bone metastases in some patients with breast cancer. However, research on prostate cancer and other solid tumors does not support the use of bisphosphonates in preventing bone metastases (**Table 1**) (127). Currently, bone-targeted drugs including denosumab, zoledronic acid, and bisphosphonates have not yet been clinically approved to prevent initial bone metastasis in patients with prostate cancer (128). Notwithstanding this fact, bisphosphonates have achieved widespread clinical recognition as a medication for managing and preventing SREs and osteoporosis related to cancer therapy (126, 129, 130). However, adjuvant bisphosphonates should not be considered a replacement for conventional anticancer treatments.

**Table 1:** Clinical trial of bisphosphonates in the treatment of bone metastases.

Medication	Tumor	Patients	Outcomes	Study	Reference
Clodronate	Breast cancer	Older postmenopausal patients	Preventing metastases	NSABP B-34	(117)
	Breast cancer	Primary breast cancer patients	Preventing metastases, extend OS and DFS		(118)
	Breast cancer	Early-stage breast cancer	Reducing SREs		(119)
	Breast cancer	Node-positive breast cancer	Negative		(120)
	Prostate cancer	Nonmetastatic prostate cancer	Negative	MRC PR04	(121)
	Prostate cancer	Metastatic prostate cancer	Negative	MRC PR05	(117)
Ibandronate	Breast cancer	Patients with postmenopausal ER+ breast cancer	Negative	TEAM-IIB	(122)
	Breast cancer	Patients with high-risk early-stage breast cancer	Negative	GAIN Study	(123)
Zoledronic acid	Breast cancer	Women with established menopause	Preventing metastases	the AZURE trial	(124)
	Breast cancer	Patients with MAF-negative tumor	Preventing metastases and reducing SREs	AZURE trial	(125)
	Breast cancer	After disease recurrence	Reducing SREs	the AZURE trial	(123)
	Prostate cancer	Patients with high-risk nonmetastatic prostate cancer	Negative	ZEUS	(126)
	Prostate cancer	Treatment-naive prostate cancer	Delay the first SREs	ZAPCA	(122)

The ASCO-CCO 2021 guidelines provide clear recommendations regarding the use of adjuvant bisphosphonate therapy for postmenopausal patients with primary breast cancer. These recommendations apply to patients eligible for adjuvant systemic therapy, regardless of their hormone receptor status or human epidermal growth factor receptor 2 status (125). The efficacy of bisphosphonates in reducing the incidence of bone metastases in patients with breast cancer was demonstrated through clinical randomized controlled trials as early as the 1990s. In a clinical trial comprising 302 patients with primary breast cancer at high risk for distal metastases, clodronate effectively mitigated the risk of bone and visceral metastases (131). However, a separate trial showed that clodronate was significantly more effective in inhibiting tumor metastasis among patients with breast cancer, specifically those aged ≥50 years, compared to the control group receiving a placebo (132). The authors attributed the variations in bisphosphonate effectiveness across various age groups to dissimilarities in estrogen levels and bone turnover mechanisms during the menopausal transition. An increasing number of clinical trials have subsequently aimed to further elucidate the specific subset of patients with breast cancer who may derive therapeutic benefits from bisphosphonate medication. A collaborative meta-analysis conducted by the Early Breast Cancer Trialists’ Collaborative Group examined the impact of adjuvant bisphosphonate treatment on breast cancer across multiple trials (133). Their study found that bisphosphonates can reduce the recurrence of breast cancer in the skeletal system and improve overall survival outcomes for individuals diagnosed with breast cancer. However, these advantages are only noticed in females who commenced the medication after the onset of menopause (133).

A recent clinical trial addressed the question of whether different bisphosphonate agents exhibit varying effectiveness in treating bone metastases in patients with breast cancer. The SWOG/Alliance/Canadian Cancer Trials Group/ECOG-ACRIN/NRG Oncology Study S0307 evaluated the effectiveness of three bisphosphonates (zoledronic acid, clodronate, and ibandronate) in patients with early-stage breast cancer (15). Neither the overall trial nor subgroup analyses showed any evidence of variations in efficacy among these three bisphosphonates in preventing bone metastatic disease. The authors also concluded that bisphosphonates should be incorporated into the treatment of postmenopausal individuals with early-stage breast cancer (15). However, administering oral ibandronate as an adjuvant treatment did not significantly improve the outcomes of patients with high-risk early-stage breast cancer who had received dose-dense chemotherapy (134). Notably, the outcomes of the TEAM-IIB study do not validate the efficacy of ibandronate in managing metastases among postmenopausal females diagnosed with estrogen receptor-positive (ER+) breast cancer (135).

To further optimize the indications for bisphosphonate treatment for bone metastasis, several clinical trials have investigated the differences in gene expression in subpopulations. The AZURE trial suggested that *MAF* can serve as a biological indicator to identify the potential beneficiary population of bisphosphonate treatment in patients with breast cancer (136). Patients with MAF-negative tumors exhibited prolonged invasive disease-free survival when treated with zoledronic acid (HR = 0.74–0.98; 95% confidence interval [CI] = 0.64–0.98) compared with those who received a placebo. However, patients with MAF positivity did not show this trend. Moreover, in a population of young menopausal patients, zoledronic acid treatment in MAF-negative patients was associated with longer overall survival (HR = 2.27; 95% CI = 1.04–4.93) and invasive disease-free survival (HR = 2.47; 95% CI = 1.23–4.97) compared with the control treatment (136).

### 5.2 Denosumab

Denosumab, a human monoclonal antibody targeting RANKL, has been approved for treating osteoporosis and giant cell tumors of the bone. It has shown greater effectiveness than bisphosphonates in managing osteoporosis (137). Furthermore, postmenopausal women may safely switch from bisphosphonate treatment to denosumab, as the latter has shown increased effectiveness in enhancing bone mineral density than the continued use of bisphosphonates (138). Moreover, compared with bisphosphonates, denosumab is a more selective inhibitor of osteoclasts, and a phase III clinical trial revealed that denosumab is more effective in preventing malignant bone metastases (**Table 2**) (142).

**Table 2:** Clinical trial of denosumab in the treatment of bone metastases.

Tumor	Patients	Outcomes	Study	Reference
Prostate cancer	Patients with castration-resistant prostate cancer	Increased bone-metastasis-free survival; delayed time to first bone metastasis		(134)
Breast cancer	Patients with stage II or III breast cancer	Negative	D-CARE	(13)
Breast cancer	Postmenopausal patients with hormone receptor-positive breast cancer	Preventing metastases and extending OS and DFS	ABCSG-18	(14)
Prostate cancer	Patients with castration-resistant prostate cancer, those with no previous bisphosphonate exposure, and those with radiographic evidence of bone metastasis	Reduced the risk of skeletal complications compared with zoledronic acid		(139)
Prostate cancer	Patients with nonmetastatic castration-resistant prostate cancer	Consistently increased bone metastasis-free survival in men, with reduced PSA doubling time		(140)
Breast cancer	Women with breast cancer and skeletal metastases	Administering ZA every 3 months was more cost-effective in reducing the risks of SRE than the monthly administration of denosumab	CALGB 70604	(141)

Denosumab does not impact overall survival in patients with castration-resistant prostate cancer. However, in a previous study, compared with placebo, denosumab significantly enhanced bone metastasis-free survival (29.5 [95% CI = 25.4–33.3] vs. 25.2 [95% CI = 22.2–29.5] months; HR = 0.85, 95% CI = 0.73–0.98, p = 0.028) and significantly delayed time to first bone metastasis (33.2 [95% CI = 29.5–38.0] vs. 29.5 [22.4–33.1] months; HR = 0.84, 95% CI = 0.71–0.98, p = 0.032) (143). In the ABCSG-18 clinical trial, which exclusively included postmenopausal patients with breast cancer (early-stage, hormone receptor-positive, nonmetastatic adenocarcinoma of the breast), the administration of denosumab as adjuvant therapy improved disease-free survival (14). Nevertheless, the results of this study do not conclusively demonstrate the efficacy of denosumab in preventing the occurrence of bone metastasis in patients with breast cancer, as the distal metastases were not histologically confirmed during the trial.

Recent clinical trial data involving more recruited individuals have also raised doubts about the effectiveness of denosumab in preventing malignant bone metastases. The absence of guideline endorsement for the use of denosumab in preventing bone metastases contrasts with the well-documented effectiveness of bisphosphonates. Following the guidelines released by ASCO-CCO in 2021, the use of adjuvant denosumab to prevent breast cancer recurrence is not recommended due to inconsistent findings in studies involving individuals with early-stage breast cancer (125). A phase III multicenter randomized controlled clinical study involving 4509 patients with breast cancer over a 5-year follow-up period found that adjuvant denosumab did not improve metastasis- and disease-free survival in patients with early-stage breast cancer (13). Additionally, there were no clinical benefits to using denosumab as an adjuvant to chemotherapy in treating nonsmall cell lung cancer (144).

The efficacy of denosumab was found to be superior to that of zoledronic acid regarding SRE prevention in patients with bone metastasis (145). Although the efficacy of denosumab as a therapy for SREs is evident, its increased application has revealed its complications. For example, the termination of denosumab treatment is associated with a rebound effect characterized by an increased likelihood of numerous spontaneous vertebral fractures (146).

### 5.3 Src inhibitors

The Src family kinases are recognized for their pivotal function in transmitting signals across diverse cellular processes and in bone metastasis formation. Suppressing Src induces a decrease in osteoclastic bone resorption, indicating its potential as a viable treatment strategy for medical disorders characterized by excessive bone resorption, including metastatic bone diseases and osteoporosis (147). Several ongoing clinical trials are assessing the effectiveness of Src kinase inhibitors, including dasatinib, KX2–391, bosutinib, AZD0424, and saracatinib (148).

According to a phase II clinical trial, dasatinib is both safe and effective in safeguarding bone integrity among patients with metastatic castration-resistant prostate cancer who had not received prior chemotherapy (149). The findings of the trial demonstrated that the administration of dasatinib induced a reduction in alkaline phosphatase levels, indicating its effectiveness in mitigating the development of new bone lesions. Nevertheless, the precise impact of dasatinib on bone lesions remains unclear. A subsequent phase III trial reported that dasatinib provided no significant benefit when combined with chemotherapy for prostate cancer (150). In particular, the READY trial demonstrated no significant difference in the survival rates of patients with metastatic castration-resistant prostate cancer who received dasatinib + docetaxel and those who only received docetaxel (21.5 [95% CI = 20.3–22.8] vs. 21.2 [95% CI = 20.0–23.4] months; HR = 0.99, 95.5% CI = 0.87–1.13, p = 0.90) (150). The investigators concluded that the addition of dasatinib to the treatment showed no significant improvement in the levels of bone turnover markers. Furthermore, they revealed that the suppressive impact on bone lesions may be attributed to docetaxel. Owing to the disappointing outcomes of several clinical trials on the effectiveness of dasatinib as a treatment for breast cancer, the feasibility of conducting additional research on its use as an independent therapy remains controversial. Alternatively, studies have suggested that the application of dasatinib may be limited to a certain group of patients exhibiting molecular expression or may be combined with other pharmaceutical medications in the future (151). Moreover, clinical trials have shown that Src enzyme inhibitors, including bosutinib, saracatinib, and KX2–391, exhibit no significant beneficial impact on bone lesions or SREs (152–154).

Although Src inhibitors have shown potential efficacy in alleviating bone resorption in certain contexts, further investigation is warranted to optimize their ability to target bones before realizing their potential advantages in clinical trials.

### 5.4 Cathepsin K inhibitors

Although preclinical investigations have established a strong correlation between cathepsin K and the occurrence of bone metastases, clinical trials have predominantly prioritized the use of cathepsin K inhibitors for treating osteoporosis and arthritis. To date, no cathepsin K inhibitor has received approval from the Food and Drug Administration. Odanacatib has emerged as the only candidate for inhibiting cathepsin K and has demonstrated considerable therapeutic efficacy in phase III clinical trials in patients with postmenopausal osteoporosis (155). A single short-term, double-blind clinical trial provisionally demonstrated the effectiveness of odanacatib in managing bone metastases in patients with breast cancer, as evidenced by one of the limited clinical trials conducted on this topic (156). The trial provided evidence regarding the inhibitory effects of odanacatib on the expression levels of osteolysis marker (urinary N-telopeptide of type I collagen corrected for creatinine) in patients with breast cancer, similar to the effects of zoledronic acid. Clinical trials on osteoporosis have recently been discontinued due to the observed increase in cardiovascular events associated with the administration of odanacatib. Consequently, the investigators have decided to refrain from allocating resources toward further research (157). Several cathepsin K inhibitors have been eliminated by researchers due to the severe adverse effects observed during clinical trials.

### 5.5 Everolimus

Everolimus is an mTOR inhibitor that suppresses the pro-osteoclast paracrine pathway of tumor cells, thereby inhibiting bone metastasis (158). In the BOLERO-2 trial, patients with bone metastasis who received everolimus showed a significant improvement in bone health, with a considerable reduction in skeletal tumor growth, compared with those in the control group (159). Furthermore, the levels of bone resorptive markers (BSAP, P1NP, and CTX) in patients treated with everolimus decreased significantly, which was attributed to the antitumor action of the drug, inducing a reduction in osteoclast function. The RADAR study suggested that everolimus can provide long-term benefits in patients with bone metastases, as long as no progression occurs during the first 8 weeks of therapy (160).

## 6 Challenges and future research

The current approach to managing bone metastases involves a combination of several therapeutic modalities. Preclinical research often lack direct applicability to real-world applications, and the use of bisphosphonates and denosumab is limited to their roles as adjuvant therapies. Nevertheless, formulating theoretical frameworks remains the primary method through which we can understand the complexity of bone metastasis. Researchers have recently found that malignant tumors expressing Cyp11a1 produce the steroid pregnenolone, which can promote the growth of bone metastases by enhancing osteoclastogenesis (161).However, researchers encounter various challenges in conducting preclinical and clinical investigations aimed at treating bone metastases.

Regarding clinical studies, the methodology used for the clinical evaluation of bone metastases differs from that used for primary tumors. Evaluating treatment efficacy in metastatic bone disease is also distinctive, focusing on specific criteria that consider both bone repair and deterioration rather than just changes in tumor size. Furthermore, assessing responses in bone metastases poses challenges due to the gradual and inconspicuous nature of the healing process. The predominant clinical investigations for bone metastases rely on imaging modalities, such as X-ray, computer tomography, magnetic resonance imaging, and Positron Emission Tomography. However, the precision of these examinations is often insufficient, limiting their capacity to identify early-stage microlesions and evaluate disease progression. Assessing multifocal bone metastases that affect both the axial and appendicular skeletons can present difficulties and inaccuracies using current imaging modalities. The absence of standardization and harmonization in clinical assessment methods across trials further complicates the precise comparison and interpretation of outcomes. This limitation hinders the ability to ascertain treatment efficacy based solely on individual study results. In the future, the use of nanoprobes holds promise for enhancing the diagnosis and assessment of bone metastases. These nanoprobes can provide extensive insights into several aspects, including anatomical structure, functional metabolism, physiological characteristics, and pathological profiles, with multimodal imaging functions for detecting ultrasmall lesions in bones and multiorgan cancer metastases (162).

The translatability and correlation between therapeutic responses observed in preclinical models and those observed in human clinical trials are often inadequate. This inadequacy can be attributed to the challenges encountered in preclinical studies on bone metastases. Notably, prevalent research models exhibit limitations in accurately replicating human bone metastases. Both animal and *in vitro* models have limitations in fully replicating the components and natural processes present in the bone metastasis microenvironment in humans. Additionally, the precise mechanisms underlying bone metastasis remain elusive, and investigations into these mechanisms are limited by the inability to observe *in vivo* processes accurately. Exploring the dynamic interactions between malignant cells and the bone niche within a living animal through intact bone requires high-resolution deep-tissue imaging, making it an essential area of research. Although high-resolution imaging protocols for deep-tissue observation exist, they are not widely available.

The current understanding of osteoclast heterogeneity is limited and requires further exploration. The osteoclast phenotype fluctuates based on various physiological and pathological conditions, and notable phenotypic differences exist among various bones. The rapid development of single-cell sequencing technology has facilitated these findings and provided a feasible approach for elucidating the mechanisms underlying bone metastasis. The use of single-cell sequencing technologies presents a viable approach for obtaining novel insights into the biological mechanisms underlying the development and advancement of bone metastasis. Moreover, single-cell methodologies have the potential to reveal the molecular mechanisms underlying the metastatic colonization of bones by accounting for significant cellular heterogeneity, plasticity, and evolutionary changes that occur during metastasis and within the complex bone microenvironment. Future in-depth studies on osteoclast heterogeneity can help reveal the mechanisms underlying bone metastasis, improve treatment targeting, and address current clinical challenges in treating bone metastasis. The combination of high-resolution single-cell and spatial technologies in a multidimensional approach has highlighted the significance of the complete cancer ecosystem, which may provide insights into the intricate mechanisms underlying bone metastasis in the future.

## Author contributions

YL: Writing – original draft, Methodology. HC: Writing – review & editing. TC: Writing – review & editing. GQ: Writing – review & editing. YH: Writing – review & editing, Supervision, Investigation, Conceptualization.
